# Changing the Antibiotic Prescribing of general practice registrars: the ChAP study protocol for a prospective controlled study of a multimodal educational intervention

**DOI:** 10.1186/s12875-016-0470-7

**Published:** 2016-06-06

**Authors:** Mieke L. van Driel, Simon Morgan, Amanda Tapley, Lawrie McArthur, Patrick McElduff, Lucy Yardley, Anthea Dallas, Laura Deckx, Katie Mulquiney, Joshua S. Davis, Andrew Davey, Kim Henderson, Paul Little, Parker J. Magin

**Affiliations:** Discipline of General Practice, School of Medicine, the University of Queensland, L8 Health Sciences Building 16/910, Royal Brisbane and Women’s Hospital, Brisbane, QLD 4029 Australia; Elermore Vale General Practice, Shop 10-13, Croudace Rd, Elermore Vale, NSW 2287 Australia; GP Synergy NSW & ACT Research and Evaluation Unit, 17 Bolton St, Newcastle, NSW 2300 Australia; Rural Clinical School, The University of Adelaide, 122 Frome Street, Adelaide, SA 5005 Australia; School of Medicine and Public Health, University of Newcastle, Newbolds Building, Corner Frith andGavey Streets, Mayfield, NSW 2304 Australia; Department of Psychology, University of Southampton, Shakleton Building, Highfield, Southampton, SO17 1BJ UK; School of Medicine, University of Notre Dame Australia, 160 Oxford St, Darlinghurst, NSW 2010 Australia; Department of Infectious Diseases, John Hunter Hospital, Lookout Rd, New Lambton Heights, NSW 2305 Australia; Global and Tropical Health Division, Menzies School of Health Research, PO Box 41096, Casuarina, NT 0811 Australia; Discipline of General Practice, School of Medicine and Public Health, University of Newcastle, Newbolds Building, Corner Frith and Gavey Streets, Mayfield, NSW 2304 Australia; Primary Care and Population Sciences Division, University of Southampton, Aldermoor Health Centre, Aldermoor Close Southampton SO16 5ST, Southampton, UK

**Keywords:** Antibacterial agents, Drug resistance, Evidence-based medicine, General practice, Graduate medical education, Physician prescribing patterns

## Abstract

**Background:**

Australian General Practitioners (GPs) are generous prescribers of antibiotics, prompting concerns including increasing antimicrobial resistance in the community. Recent data show that GPs in vocational training have prescribing patterns comparable with the high prescribing rate of their established GP supervisors. Evidence-based guidelines consistently advise that antibiotics are not indicated for uncomplicated upper respiratory tract infections (URTI) and are rarely indicated for acute bronchitis. A number of interventions have been trialled to promote rational antibiotic prescribing by established GPs (with variable effectiveness), but the impact of such interventions in a training setting is unclear. We hypothesise that intervening while early-career GPs are still developing their practice patterns and prescribing habits will result in better adherence to evidence-based guidelines as manifested by lower antibiotic prescribing rates for URTIs and acute bronchitis.

**Methods/design:**

The intervention consists of two online modules, a face-to-face workshop for GP trainees, a face-to-face workshop for their supervisors and encouragement for the trainee-supervisor dyad to include a case-based discussion of evidence-based antibiotic prescribing in their weekly one-on-one teaching meetings.

We will use a non-randomised, non-equivalent control group design to assess the impact on antibiotic prescribing for acute upper respiratory infections and acute bronchitis by GP trainees in vocational training.

**Discussion:**

Early-career GPs who are still developing their clinical practice and prescribing habits are an underutilized target-group for interventions to curb the growth of antimicrobial resistance in the community. Interventions that are embedded into existing training programs or are linked to continuing professional development have potential to increase the impact of existing interventions at limited additional cost.

**Trial registration:**

Australian New Zealand Clinical Trials Registry, ACTRN12614001209684 (registered 17/11/2014).

**Electronic supplementary material:**

The online version of this article (doi:10.1186/s12875-016-0470-7) contains supplementary material, which is available to authorized users.

## Background

Antimicrobial resistance is a serious and increasing threat to global public health and it is linked to the over-prescription of antibiotics [1, 2]. The majority of antibiotics consumed by humans are prescribed in primary care [2, 3], and they are often prescribed in conditions for which there is no evidence of benefit [4, 5]. For example, initial use of antibiotics is not of benefit in acute upper respiratory tract infections (URTIs) and is associated with an increase in adverse effects [6]. For acute bronchitis, antibiotics are associated with limited benefits of doubtful clinical significance [4].

Evidence-based therapeutic guidelines are widely used in general practice. The *“Australian Therapeutic Guidelines: Antibiotic v15”* for example recommend symptomatic (non-antibiotic) treatment for acute uncomplicated URTIs and advise that acute bronchitis is most often viral and usually does not require antibiotic therapy [7]. Availability and knowledge of guidelines, however, does not guarantee adherence which is reflected in the level of over-prescribing of antibiotics [8]. Data from Australian general practitioners (GPs) in training showed that antibiotics are prescribed in 22 % of presentations for URTIs, and 73 % of bronchitis/bronchiolitis [9]. These prescription rates are comparable to those of established GPs. This clear overprescribing of antibiotics suggests that more effort needs to be directed towards changing the prescribing habits of clinicians.

In high-income countries, public campaigns at a regional and national level using a variety of multimedia platforms, have been directed at the public, child-care staff and health professionals [10]. Most campaigns also encouraged the use of guidelines in the components that targeted prescribers. Results from some multifaceted campaigns repeated over several years suggest a positive effect on antibiotic use, but others seem to have had little if any effect [10]. The means by which the prescribing patterns of GPs might be influenced is still the focus of debate. In primary care, a variety of interventions aimed at influencing individual doctors’ prescribing have been studied, including audit and feedback [11], small group meetings [12], and academic detailing [13]. Systematic reviews have established that in primary care and ambulatory practice, multi-faceted interventions that include combinations of the above approaches [14] and active, rather than passive, interventions [15] are the most successful in reducing antibiotic prescribing for inappropriate indications.

Unfortunately, the size of the effect of these approaches has been limited. For instance, a UK randomised control trial incorporated the above principles in an intervention to reduce antibiotic prescribing by GPs [16]. This produced a modest decrease in antibiotic prescribing of 4.2 % in the intervention relative to the control practices. Moreover, the intervention was expensive and cost £2923 per practice – several times the estimated annual savings in antibiotics cost. The European GRACE consortium, however, showed an encouraging and likely more affordable way to achieve meaningful reductions in antibiotic prescribing for lower respiratory tract infections [17]. This intervention used online training modules on communication skills and on the use of C-reactive protein (CRP) to distinguish bacterial from presumed viral infections. Both communication training and introduction of CRP separately resulted in a reduction of antibiotic prescribing, with the combination achieving the largest effect (communication intervention risk ratio: 0 · 68; CRP: 0 · 53; combined intervention: 0 · 38) [18].

It is plausible that interventions targeted at an early, receptive stage of GPs’ careers might be more efficacious. Early-career GPs may be a singularly appropriate target for intervention. They are at a stage of their clinical careers where their prescribing patterns are unlikely to be firmly established and are thus more amenable to modification. Unlike hospital-based junior doctors, early-career GPs are working in the clinical environment where they will spend the rest of their careers, so education regarding long-term prescribing practices will be contextually relevant. Because experienced clinicians have already established consulting and antibiotic prescribing habits that have been shown to be resistant to change [19], we hypothesise that focused educational interventions that target GPs in training whom are still developing their own practice “style” and prescribing “habits”, could be more effective in improving adherence to evidence-based guidelines.

## Methods/design

The ChAP study (Changing the Antibiotic Prescribing of general practice registrars) aims to improve the antibiotic prescribing of early-career GPs. In this study we propose to develop an Australian educational intervention based on components of the INternet TRaining for antibiOtic use (INTRO) intervention, developed in Europe [17]. To assess the effectiveness of the intervention, ongoing quantitative data collection from the Registrar Clinical Encounters in Training (ReCEnT) Study will be utilized [20]. We will evaluate the intervention’s impact on antibiotic prescribing of early-career GPs for two classifications of respiratory tract infections (URTI and acute bronchitis/bronchiolitis).

### Study design

We will use a non-randomised non-equivalent control group design. Data for the analyses will be collected during the six-monthly rounds of ReCEnT data collection. The ReCEnT study is an ongoing prospective multi-site cohort study of GP registrars/trainees' consultations in five of Australia’s 17 Australian GP regional training providers [20]. ReCEnT longitudinally documents the nature and association of consultation-based clinical and educational experiences of early-career GPs (GP trainees). GP trainees record details of 60 consecutive consultations at approximately the mid-point of three six-month (full-time equivalent) terms based in general practices. Details recorded include patient demographics, diagnoses/problems managed, medications prescribed, investigations ordered, and assistance sought during the consultation (including supervisor advice and use of guidelines).

### Outcome measures

#### Primary outcomes

The primary outcomes will be

- Change in proportion of consultations (from pre-intervention to post-intervention ReCEnT data collection periods) for URTIs in which antibiotics are prescribed
- Change in proportion of consultations (from pre-intervention to post-intervention ReCEnT data collection periods) for bronchitis/bronchiolitis in which antibiotics are prescribed.

#### Secondary outcomes

Secondary outcomes will be

- Change in proportion of consultations (from pre-intervention to post-intervention ReCEnT data collection periods) for bronchitis/bronchiolitis and URTI (combined) in which antibiotics are prescribed.
- Change in proportion of consultations (from pre-intervention to post-intervention ReCEnT data collection periods) for sore throat in which antibiotics are prescribed
- Change in proportion of consultations (from pre-intervention to post-intervention ReCEnT data collection periods) for acute sinusitis in which antibiotics are prescribed
- Change in proportion of consultations (from pre-intervention to post-intervention ReCEnT data collection periods) for acute otitis media in which antibiotics are prescribed

Respiratory tract infections will be defined by International Classification of Primary Care, 2nd Edition (ICPC-2) disease diagnostic codes R74 (URTI) and R78 (bronchitis/bronchiolitis) [21].

We will define acute sore throat as the ICPC-2 codes R72 (strep throat), R76 (tonsillitis, acute), R74008 (pharyngitis, acute), R74006 (infection, throat), R74017 (pharyngitis) and R21005 (sore throat). Codes R72001 (scarlet fever), R72003 (scarlatina) and R72004 (scarlet fever) will be excluded from the analysis, as antibiotics are recommended for all cases of these conditions in the authoritative Australian guidelines [22].

We will define acute sinusitis as those presentations coded as R75 (excluding R75003 and R75004 as these are codes for chronic and allergic sinusitis respectively).

Acute otitis media will be defined as those presentations coded as H71 (excluding H71008, H710018 and H710019 as these are codes for ear inflammation, abscess and mastoiditis).

### Setting

The intervention will be delivered once to GP trainees in two Regional Training Providers (RTPs). GP trainees in three control training providers will receive usual educational activities. These are five of the 17 RTPs in the Australian general practice vocational training program. The demographics of the GP trainees in the intervention group are broadly comparable to those of the control training providers.

### The intervention

**Fig. 1:**
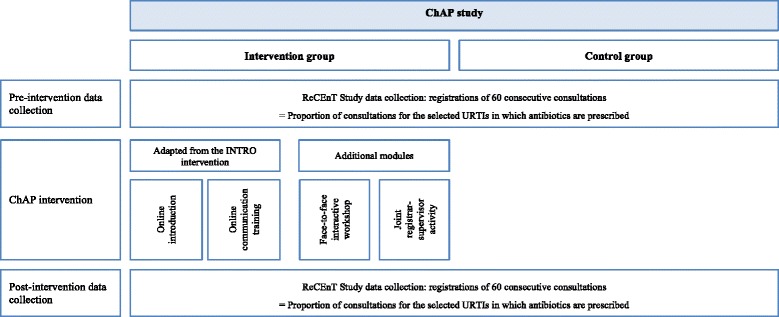
Study design. ChAP study, Changing the Antibiotic Prescribing of general practice registrars; ReCEnT study, Registrar Clinical Encounters in Training; URTIs, Upper Respiratory Tract Infections (upper respiratory tract infections and bronchitis/bronchiolitis); INTRO, INternet TRaining for antibiOtic use

The intervention in the ChAP study consists of four components. The first three i.e. access to an online introduction module, an online communication training module, and an interactive workshop, are delivered separately to GP trainees and to supervisors. The fourth is an optional joint GP trainee-supervisor activity, which comprises a case-based discussion of evidence-based antibiotic prescribing for each trainee-supervisor dyad in their regular weekly one-on-one teaching meetings. An overview of the study design and content of the intervention is provided in Fig. 1.

#### INTRO modules

The first two components (online introduction and communication training modules) are based on the INTRO intervention. INTRO is part of a European multi-country study (GRACE consortium) aimed at reducing inappropriate antibiotic prescribing for lower respiratory tract infections in primary care [17]. One of the main advantages of INTRO is that it is a brief and cost-effective intervention. The introduction module discusses the need for reduced antibiotic prescribing and the effect of over-prescription on the health care system, patients and GPs. It covers the epidemiology of URTIs in primary care, Australian and international antibiotic use and antimicrobial resistance, and the evidence-base of current clinical guidelines. The communication training module consists of videos showing how a GP can use communication skills to elicit patient concerns and expectations, discuss the pros and cons of antibiotic treatment and the natural course of respiratory tract infections, whilst maintaining patient satisfaction. The communication training module is supported by a patient booklet. These two modules were adapted for the Australian context with particular reference to the Australian Therapeutic Guidelines: Antibiotic. Version 15 (version 2013): TG [7].

The online modules will be made available to GP trainees to work through individually at a time that best suits them. They will be given a timeframe in which to complete the modules. A face-to-face workshop will be scheduled after completion of the electronic modules in order to maximise the potential for effectiveness.

The INTRO modules will also be made available to the trainee’s supervisors in the two intervention RTPs, again prior to scheduled separate supervisor workshops (see below).

#### Workshops

The workshop component consists of a 1.5 h interactive workshop, scheduled as part of the standard training program for GP trainees and delivered at the premises of their RTP. We will use data on GP trainees' prescribing collected in the ReCEnT project to contextualise and reinforce the practical relevance and importance of the educational message (the ReCEnT data will be that of the workshop attendee trainees and their more senior colleagues who have been collecting ReCEnT data since 2010) [20]. During the workshop sessions, GP trainees will be asked to reflect on their group’s prescribing data and by means of clinical cases work through the main principles of rational prescribing of antibiotics informed by the authorative Australian guideline on antibiotic use (eTG). During the workshop there will be room for practising the communication skills presented in the online modules.

There will be ‘content’ discussion in the workshops, which will expand upon and explore some of the issues covered in the online modules. This discussion will present URTIs and acute bronchitis as exemplars of respiratory tract infections for which antibiotics are rarely indicated. Discussion will focus on these clinical syndromes, but the tenets of rational prescribing will also be applied to other non-pneumonia respiratory tract infections for which antibiotics have very limited indications (sore throat, acute otitis media, acute sinusitis).

Three underlying principles will inform the approach presented in the discussion: Firstly, that the default position for non-pneumonia respiratory tract infections is to *not* prescribe antibiotics. Deviation from this principle in any particular case requires careful consideration of the rationale for prescribing in that individual patient’s circumstance. Secondly, treatment of non-pneumonia respiratory tract infections should be on syndromic rather than aetiological basis. Delineation of viral versus bacterial aetiology is difficult and non-contributory and does not reflect current understanding of the complex interplay of bacterial and viral pathogens [23, 24]. A syndromic approach to management reflects the empirical evidence for treatment in the area [5, 25–27]. Thirdly, that the clinical science of consultations for non-pneumonia respiratory tract infections may be straightforward but managing patient expectations may be complex and require advanced communication skills, i.e. the sophistication of consultation techniques employed should reflect the biopsychosocial complexity of the consultation rather than the biological complexity of the clinical presentation.

The workshop content will be constructed by the research team of GPs, GP vocational training educators, academic GPs and an infectious diseases physician/researcher. The process will be informed by the current literature in the area and our recent work in early-career GPs' antibiotic prescribing – the prevalence and associations of antibiotic prescribing for URTI and acute bronchitis/bronchiolitis and the qualitative experiences of GP trainees in managing URTI and acute bronchitis [9, 28].

The same workshop will be delivered separately to the supervisors in the two intervention RTPs.

#### Joint trainee-supervisor activity

Each trainee-supervisor dyad will be encouraged to include a case-based discussion of evidence-based antibiotic prescribing in their regular weekly one-on-one teaching meetings. The supervisor will be offered a set of three or four structured cases to include in the meeting. This activity will be optional as the content of trainee-supervisor weekly meetings is at the discretion of the supervisors and trainees rather than the RTP.

The rationale for supplementing the educational intervention with supervisor educational activities was that our previous research suggested that the prescribing patterns (role-modelling) of supervisors and the ‘apprenticeship’ model of the trainee-supervisor relationship are drivers of non-rational antibiotic prescribing [9, 28].

### Recruitment

All GP trainees at the five training providers complete ReCEnT data collection in each of their training terms. This is part of their training program and includes reflection on practice and future training directions via detailed feedback [29]. The majority of GP trainees consent to the data being used for research purposes. The intervention will be conducted as part of their training program.

### Sample size calculation

It is expected that the intervention group will consist of approximately 130 GP trainees sourced from two intervention training providers and the control group of approximately 160 GP trainees from the control training providers. Each of these GP trainees will provide data on an average of four URTI consultations before the intervention and four URTI consultations after the intervention [9]. This will provide the study with approximately 80 % power to find an absolute reduction of 10 % in antibiotic prescribing for URTIs in the intervention group compared with the control group. This sample size calculation takes into account an inflation factor of 1.9 derived from an intracluster correlation of 0.27 from a similar study [12] and an anticipated proportion of consultations for URTI of 8 % and expected pre-intervention rate of antibiotic prescribing of 22 % as shown in the ReCEnT study [9].

### Data collection and analysis

Change in antibiotic prescribing will be assessed via ReCEnT data pre- and post-intervention. The changes in GP trainees’ prescribing will be compared with the changes of GP trainees of the two regional training providers who are not receiving the intervention (control group).

The principal analysis will be logistic regression within a Generalised Estimating Equation framework to adjust for clustering of observations within GP trainees. The unit of analysis will be consultations involving a respiratory tract infection and the outcome factor will be antibiotics prescribed (dichotomous). Independent variables in the model will be treatment group (intervention/control), time (before/after) and an interaction term of treatment group by time. The p-value of the interaction term will be used to determine statistical significance.

The dependent variables in regression models will be consultations in which antibiotics are prescribed (for URTI and acute bronchitis/bronchiolitis separately as the primary outcomes, and for URTI plus acute bronchitis/bronchiolitis (combined), acute otitis media, acute sinusitis and sore throat as the secondary outcomes).

We will also consider trainee, patient, practice and consultation related factors in the regression analyses. GP trainee factors will be age, gender, training term, place of medical qualification (Australia/international), and full-time/part-time status. Patient factors will be age, gender, Indigenous (Aboriginal or Torres Strait Islander) status, Non-English-speaking background status, whether new patient to the practice, and whether a new patient to the GP trainee. Practice factors will be rurality/urbanicity, socioeconomic status of the practice location, and practice size (number of GPs). Practice postcode will be used to assign the Australian Standard Geographical Classification-Remoteness Area (ASGC-RA) classification (the degree of rurality) of the practice location [30] and to define the practice location’s Socioeconomic Index for Area (SEIFA) Relative Index of Disadvantage [31]. Consultation factors will include the number of diagnoses/problems dealt with during the consultation, if the problem was a new one, and if the GP trainee sought clinical assistance during the consultation (from their supervisor/trainer, from a specialist, or from electronic or hard-copy resources).

Primary analyses will be by intention to treat (data of all GP trainees in the intervention group will be analysed irrespective of whether they attended the intervention workshop). We will also analyse the per protocol data (of only those GP trainees who attend the workshop) and compare these with the intention to treat analysis.

### Qualitative study

In addition, GP trainees and supervisors who participated in the intervention, will be invited to take part in a telephone interview after the full ChAP intervention has been delivered. Interview questions will focus on the acceptability and utility of the intervention components, the elements that changed practice, and suggestions for improvement. All interviews will be recorded digitally and transcribed verbatim. Two researchers will independently code the interviews, compare their coding and discuss discrepancies. Interview data will be analysed using thematic analysis [32]. An iterative process will be used to develop the final thematic framework.

### Funding and ethics

This study is funded by a competitive research grant of the Therapeutic Guidelines Limited and the Royal Australian College of General Practitioners. Funding was awarded after a peer-review process assessing the importance, scientific quality, and originality of the study. The intervention is registered in the Australian New Zealand Clinical Trials Registry (ACTRN12614001209684).

The wider ReCEnT project is funded by the five participating Regional Training Providers. These organisations are funded by General Practice Education and Training, an Australian Commonwealth Government initiative.

This study was approved by the Human Research Ethics Committee from the University of Newcastle (H-2009-0323) and The University of Queensland (2014000743). Confidentiality will be ensured by the current practice regarding ReCEnT data. All ReCEnT data collection is de-identified. Registrations can only be linked to the same early-career GP through a coding system. The key to this code is not known to the investigators and kept separately in a locked filing cabinet and password protected computer file at the regional training providers where the data are collected.

Following the completion of the study, the intervention will be offered to the control training providers and be made available to other Australian regional training providers (Additional file 1).

## Discussion

Experienced clinicians have already established distinct consulting and prescribing habits, which have been shown to be resistant to change [19]. Interventions on antibiotic prescribing have a modest effect [16]. In the proposed study we target educational interventions to early-career GPs when they are still developing their own prescribing “style” and “habits”. This approach could be more effective in improving adherence to evidence-based guidelines.

Delivering educational interventions to early-career GPs rather than established GPs may also be a more efficient and sustainable way to educate prescribers about evidence-based use of antibiotics. Australian early-career GPs are engaged in an educational program during release days from their practice, in parallel with their practice-based training. Face-to-face educational activities, as well as access to web-based support, could be delivered within the context of their existing educational program. The infrastructure to deliver the intervention already exists. Use of this infrastructure would avoid the organisational demands of staging individual practice seminars and setting up dedicated web-based support systems. This study will produce a template for an educational model targeting clinicians/prescribers in the early stages of their careers. The impact of the educational intervention will be evaluated by the ReCEnT study, thereby allowing an efficient integration with a concurrent education and research project [19]. Moreover, whilst this intervention focuses on adherence to guidelines for rational prescribing of antibiotics in respiratory tract infections in the general practice setting, it has the potential to serve as a generic model to enhance uptake of therapeutic guideline recommendations in general.

One limitation of the study is that it will not be possible to ascertain the appropriateness of prescription of antibiotics in any single clinical encounter. For example, in bronchitis where antibiotic prescription is discouraged for the great majority of patients, antibiotics may be appropriate for those with severe co-morbid conditions and immunosuppression [7]. However, overall antibiotic prescribing for non-pneumonia acute respiratory tract infections, and for each individual condition, is still a robust measure of appropriate prescribing at the macro-level and has been used in numerous previous studies [12–17].

A further consideration is that prescription is not necessarily followed by dispensing (filling the script), or even consumption of antibiotics by the patient. In our study we will ascertain prescription of antibiotics only. An advantage of our methodology is that we also capture the indication for prescribing, as compared with other studies, which report total, non-disease-specific dispensing [16]. As the focus of this intervention is rational prescribing of antibiotics in acute URTIs and acute bronchitis/bronchiolitis, measuring indication-specific prescribing will provide the most appropriate indicator of the impact of the intervention. If the intervention is successful, further studies could assess the impact of a reduction in prescribing on overall dispensing and consumption of antibiotics and antimicrobial resistance rates.

The multimodal and multifaceted model developed from the ChAP study can also be used in programs targeting established GPs and inform effective interventions to curb the growth of antimicrobial resistance through rational prescribing of an increasingly precious resource.

## Abbreviations

ASGC-RA, Australian Standard Geographical Classification-Remoteness Area; ChAP, Changing the Antibiotic Prescribing of general practice registrars; CRP, C-reactive protein; GP, general practitioners; ICPC-2, International Classification of Primary Care, 2nd Edition; INTRO, INternet TRaining for antibiOtic use; ReCEnT, Registrar Clinical Encounters in Training; RTP, regional training provider; SEIFA, Socioeconomic Index for Area; URTIs, upper respiratory tract infections

## Additional file

Additional file 1: TIDieR (Template for Intervention Description and Replication) checklist. (PDF 120 kb)
